# Metathesis access to monocyclic iminocyclitol-based therapeutic agents

**DOI:** 10.3762/bjoc.7.81

**Published:** 2011-05-27

**Authors:** Ileana Dragutan, Valerian Dragutan, Carmen Mitan, Hermanus CM Vosloo, Lionel Delaude, Albert Demonceau

**Affiliations:** 1 Institute of Organic Chemistry, Romanian Academy, 202B Spl. Independentei, P.O. Box 35-108, Bucharest 060023, Romania; 2 School of Physical and Chemical Sciences, North-West University, Hoffman Street, Potchefstroom 2520, South Africa; 3 Macromolecular Chemistry and Organic Catalysis, Institute of Chemistry (B6a), University of Liège, Sart Tilman, Liège 4000, Belgium

**Keywords:** azasugars, iminocyclitols, natural products, olefin metathesis, Ru-alkylidene catalysts

## Abstract

By focusing on recent developments on natural and non-natural azasugars (iminocyclitols), this review bolsters the case for the role of olefin metathesis reactions (RCM, CM) as key transformations in the multistep syntheses of pyrrolidine-, piperidine- and azepane-based iminocyclitols, as important therapeutic agents against a range of common diseases and as tools for studying metabolic disorders. Considerable improvements brought about by introduction of one or more metathesis steps are outlined, with emphasis on the exquisite steric control and atom-economical outcome of the overall process. The comparative performance of several established metathesis catalysts is also highlighted.

## Review

### Introduction

Synthetic and natural polyhydroxylated N-heterocyclic compounds (pyrrolidines, piperidines, piperazines, indolizines, etc., and higher homologues), commonly referred to as azasugars, iminosugars or iminocyclitols, can be considered as carbohydrate mimics in which the endocyclic oxygen atom of sugars has been replaced by an imino group. This vast and highly diversified class has attracted considerable interest due to the remarkable biological profile shown by many of its members which has been detailed in a number of excellent books and reviews [1–12]. Natural iminosugars (i.e., alkaloids mimicking the structures of sugars, widespread in many plants or microorganisms) [12–15], as well as non-natural counterparts, are becoming important leads for drug development in a variety of therapeutic areas, e.g., treatment of cancer [16–20], glycosphingolipid storage disorders [21–22], Gaucher’s disease [23], type-II diabetes [24–26], other metabolic disorders [10], and viral diseases [27–28] such as HIV [29–30] and hepatitis B [31–32] and C [27,33]. Some such products have been already marketed, such as *N*-hydroxyethyl-1-deoxynojirimycin (Miglitol) and *N*-butyl-1-deoxynojirimycin (Miglustat) which are active against type-II diabetes and Gaucher’s disease, respectively.

The broad biological activity of iminocyclitols has attracted growing interest in the synthesis of naturally occurring iminocyclitols and in their structural modification. Consequently, efficient and stereoselective synthetic routes have been developed, often starting from an inexpensive chiral-pool of precursors, in particular carbohydrates that share structural features with iminocyclitols. The main hurdles in this approach are the singling out of only one of the hydroxy groups in the open carbohydrate-derived intermediate, converting this hydroxy group into an amino group, and intramolecularly closing this intermediate [8,34–36]. Because of the high density of functional groups, proper protection throughout the overall synthesis scheme is an important feature that must be considered carefully, with full deprotection occurring in the final step.

**Scheme 1:**
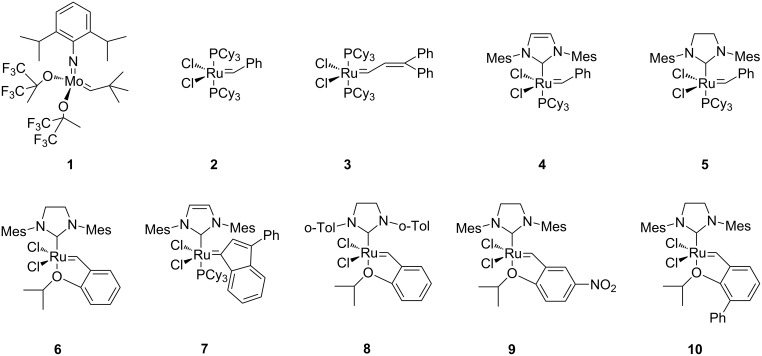
Well-defined Mo- and Ru-alkylidene metathesis catalysts.

With the advent of well-defined Mo- and Ru-alkylidene metathesis catalysts (e.g., **1**–**10**; Scheme 1) [37–47] the RCM strategy was immediately recognized as central to success in the flexible construction of N-heterocyclic compounds, including azasugars. Moreover, the metathesis approach to azasugars has greatly benefited from the vast synthetic experience acquired in RCM preparation of a host of heterocycles. Any RCM-based protocol to iminocyclitols implies three crucial stages: (i) discovery of a route to obtain stereoselectively, starting from an ordinary substrate, the N-containing prerequisite diene precursor; (ii) RCM cyclization of this diene, with an active catalyst, to access the core cyclic olefin; and (iii) dihydroxylation of the endocyclic double bond in a highly diastereoselective manner to form the target product.

In comparison to the traditional, lengthier syntheses of iminocyclitols, the metathesis approach has emerged as a highly advantageous method in terms of atom economy. However, before carrying out the RCM reaction, the basic amino group (incompatible with most metathesis catalysts because of chelation to the metal center) [48] must either be protected (as *N*–Boc, *N*–Cbz, etc.), masked by incorporation into a cleavable heteroatom-containing cycle (oxazolidine, cyclic ketal, etc.), or deactivated by conversion into amide or carbamate functions. Due to these protective groups even metathesis catalysts sensitive to functionalities can act efficiently under reaction conditions where an adequate balance between activity/stability factors has been met. In addition, the reaction conditions (temperature, solvents) currently employed in olefin metathesis reactions can be productively transferred to the metathesis steps of iminocyclitols synthesis.

By surveying the field of recent azasugar developments, this review focuses on metathesis reactions (mainly RCM, CM) as essential transformations in the multistep synthesis of monocyclic iminocyclitols, while also discussing the successes and failures in effecting the above mentioned three critical stages. New perspectives may open up for practitioners of both glyco- and metathesis chemistry involved in the synthesis and development of iminocyclitols.

### Pyrrolidine-based iminocyclitols

Recently, pyrrolidine-based iminocyclitols have assumed increasing importance and some of them have achieved even higher biological significance than the established six-membered piperidine deoxynojirimycin (DNJ) and deoxygalactonojirimycin (DGJ). Five-membered iminocyclitols possessing N-alkyl and C_1_-alkyl substituents form a class of potent antiviral compounds active, e.g., against hepatitis B virus (HBV), hepatitis C virus (HCV), and human immunodeficiency virus (HIV) [49–52].

**Scheme 2:**
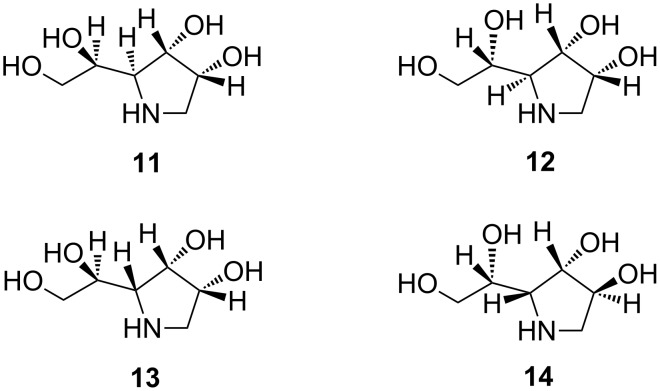
Representative pyrrolidine-based iminocyclitols.

Biological activity of this family of iminocyclitols is dictated by the stereochemistry at all carbon atoms of the pyrrolidine ring system which can adopt either a manno or a galacto conformation, therefore inhibiting either α-mannosidases (e.g., **11**–**13**) or α-galactosidases (e.g., **14**) (Scheme 2). A characteristic feature in **11**–**14** is the presence of a 1,2-dihydroxyethyl side chain.

**Scheme 3:**
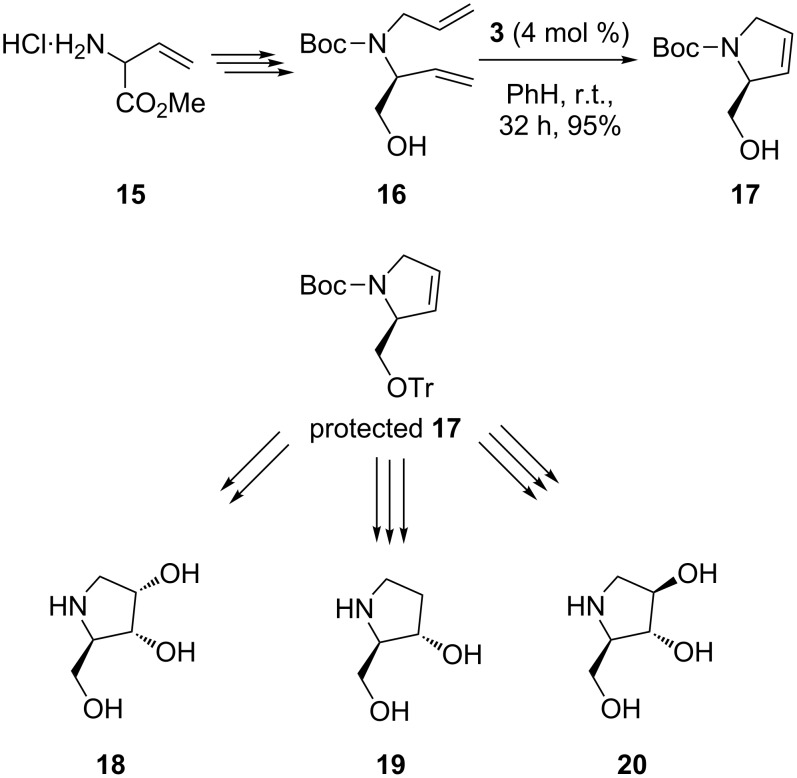
Synthesis of (±)-(2R*,3R*,4S*)-2-hydroxymethylpyrrolidin-3,4-diol (**18**), (±)-2-hydroxymethylpyrrolidin-3-ol (**19**) and (±)-(2R*,3R*,4R*)-2-hydroxymethylpyrrolidin-3,4-diol (**20**).

Following the RCM-based strategy (vide supra), an elegant and quite flexible synthesis of five-membered iminocyclitols was achieved by Huwe and Blechert as early as 1997 [53]. Starting from (±)-vinylglycine methyl ester **15** and going successively via amino protection (Cbz) and ester group reduction (LiBH_4_), a protected racemic diene **16** was obtained; RCM cyclization of the latter using the Grubbs catalyst Cl_2_(PCy_3_)_2_Ru=CH–CH=CPh_2_ (**3**) led to the racemic dehydroprolinol derivative **17** in high yield. Subsequent O-protection with trityl chloride and dihydroxylation (with OsO_4_ or stereoselective epoxidation followed by regioselective epoxide opening) produced the racemic iminocyclitols (**18**–**20**) in good overall yields (Scheme 3).

**Scheme 4:**
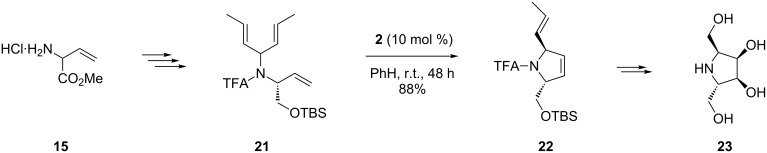
Synthesis of enantiopure iminocyclitol (−)-(2*S*,3*R*,4*S*,5*S*)-2,5-dihydroxymethylpyrrolidin-3,4-diol (**23**).

In addition, Blechert showed that this method was more adaptable as it could also yield enantiopure **18**–**20**, provided that racemization was avoided both during ester group reduction and the subsequent steps. By a similar approach (Scheme 4), these authors also obtained the enantiopure homoiminocyclitol (−)-(2*S*,3*R*,4*S*,5*S*)-2,5-dihydroxymethylpyrrolidin-3,4-diol (**23**). Starting from the same racemic vinyl glycine methyl ester and introducing enzymatic resolution in the ester reduction step, enantiopure (+)-**21** was obtained. 1st-generation Grubbs catalyst was used for the RCM of (+)-**21**. It should be noted that the yield of (+)-**22** in RCM (10 mol % **2**, in benzene) was temperature dependent (88% at room temperature and 98% at 80 °C). Further stereocontrolled dihydroxylation with simultaneous deprotection of (+)-**22** gave the final product (−)-**23** in good yield.

**Scheme 5:**
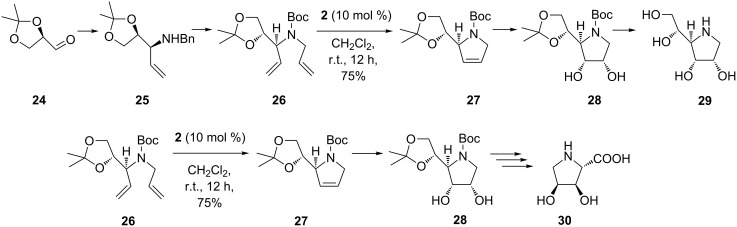
Synthesis of 1,4-dideoxy-1,4-imino-D-allitol (**29**) and formal synthesis of (2*S*,3*R*,4*S*)-3,4-dihydroxyproline (**30**).

In an interesting work by Rao and co-workers [54] a Grignard reaction was employed to design the diene with desired stereochemistry for the synthesis of 1,4-dideoxy-1,4-imino-D-allitol (**29**) and the formal synthesis of (2*S*,3*R*,4*S*)-3,4-dihydroxyproline (**30**) (Scheme 5). According to their methodology, (*R*)-2,3-*O*-isopropylidene-D-glyceraldehyde (**24**) was treated in a one-pot reaction with benzylamine and then subjected to Grignard addition with vinylmagnesium bromide to provide the alkene **25** as a single diastereomer. The nitrogen atom in **25** was then Boc-protected, debenzylated, and allylated to give the diene **26**. RCM of the latter with 1st-generation Grubbs catalyst (10 mol % **2**, in dichloromethane, at room temperature) provided the pyrrole scaffold **27**. Subsequent stereoselective dihydroxylation (OsO_4_ and 4-methylmorpholine *N*-oxide (NMO) in acetone) to yield **28** and final deprotection (MeOH–HCl) gave the imino-D-allitol **29** as the HCl salt. Formal synthesis of (2*S*,3*R*,4*S*)-3,4-dihydroxyproline (**30**) starting from **24** was carried identically by RCM to afford **27** and subsequent conversion of **28** to **30** was achieved in several steps via the Fleet protocol [55].

The tandem RCM/dihydroxylation sequence was also applied by Davis et al. in the synthesis of (−)-2,3-*trans*-3,4-*cis*-dihydroxyproline. In this case, an α-amino aldehyde, prepared by addition of a 1,3-dithiane to a chiral *N*-sulfinyl imine, was used as the chiral starting material [56]. Syntheses of 1,4-dideoxy-1,4-imino derivatives of L-allitol and D-talitol were also accomplished following a similar RCM methodology by Rao and co-workers [57].

**Scheme 6:**
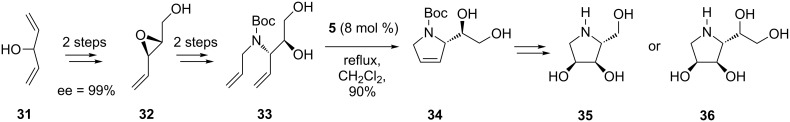
Synthesis of iminocyclitols **35** and **36**.

1,4-Dideoxy-1,4-imino-D-ribitol (**35**), known as (+)-DRB, is a potent inhibitor of glucosidases and of eukaryotic DNA polymerases. Its synthesis, as well as that of its dihydroxylated homologue **36**, features as the key step five-membered ring formation via RCM induced by the 2nd-generation Grubbs catalyst **5** (Scheme 6) [58].

**Scheme 7:**
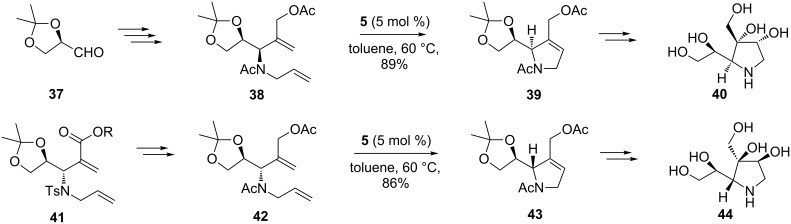
Total synthesis of iminocyclitols **40** and **44**.

A further contribution to new pyrrolidine-based azasugars, characteristically having 1,2-dihydroxyethyl side chains and a quaternary C-atom possessing a hydroxy and a hydroxymethyl group, was made by Vankar et al. [59] (Scheme 7). By ingeniously combining a Baylis–Hillman addition with RCM as the key steps, they obtained, stereoselectively and in high yields, 1,4-dideoxy-1,4-iminohexitols **40** and **44** which showed moderate inhibition of β-galactosidase, and α-galacto- and α-mannosidases, respectively. It should be noted that diene **38** did not cyclize in the presence of 1st-generation Grubbs catalyst, even in refluxing toluene, whereas 2nd-generation Grubbs catalyst afforded (in toluene, at 60 °C) the cyclic products **39** and **43** in 89% and 86% yields, respectively. Interestingly, Upjohn dihydroxylation of **39** or **43** (OsO_4_, NMO, acetone/H_2_O/*t*-BuOH; HCl, MeOH; Ac_2_O, Et_3_N, DMAP) gave only one diastereomeric diol, because the bulky acetonide group blocks the β-face of the trisubstituted double bond of the pyrrolidine ring and is thus responsible for the high diastereoselectivity.

**Scheme 8:**
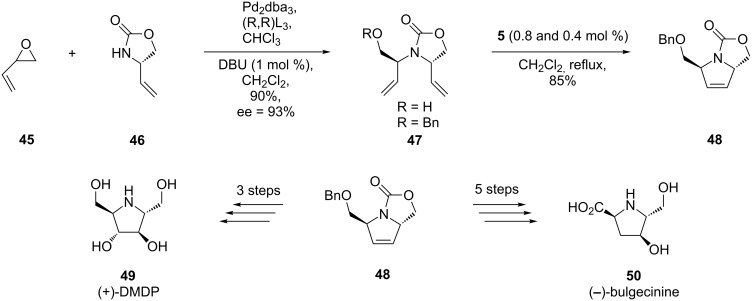
Synthesis of 2,5-dideoxy-2,5-imino-D-mannitol [(+)-DMDP] (**49**) and (−)-bulgecinine **(50**).

A metathesis approach has elegantly been used by Trost et al. for the total synthesis of 2,5-dideoxy-2,5-imino-D-mannitol [(+)-DMDP], **49**, (−)-bulgecinine, **50**, and (+)-broussonetine G, **53** [60–61]. The crucial intermediate, the protected annulated oxazolone **48**, resulted from RCM (2nd-generation Grubbs catalyst) of the imino diene **47** (previously produced by a Pd-catalyzed asymmetric transformation). The following three or five steps, respectively, including the enantioselective dihydroxylation of the RCM product **48**, occurred smoothly to produce the (+)-DMDP (**49**) and (−)-bulgecinine (**50**) (Scheme 8).

**Scheme 9:**
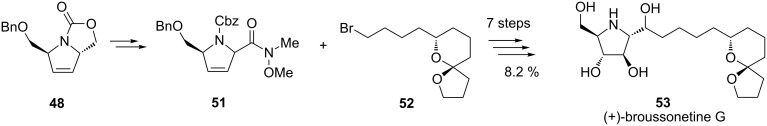
Synthesis of (+)-broussonetine G (**53**).

The starting point in the synthesis of (+)-broussonetine G, **53**, was the same annulated oxazolone **48** which, after conversion into the Weinreb amide **51**, was coupled with the alkyl bromide substituted spiro compound **52** (Scheme 9).

**Scheme 10:**
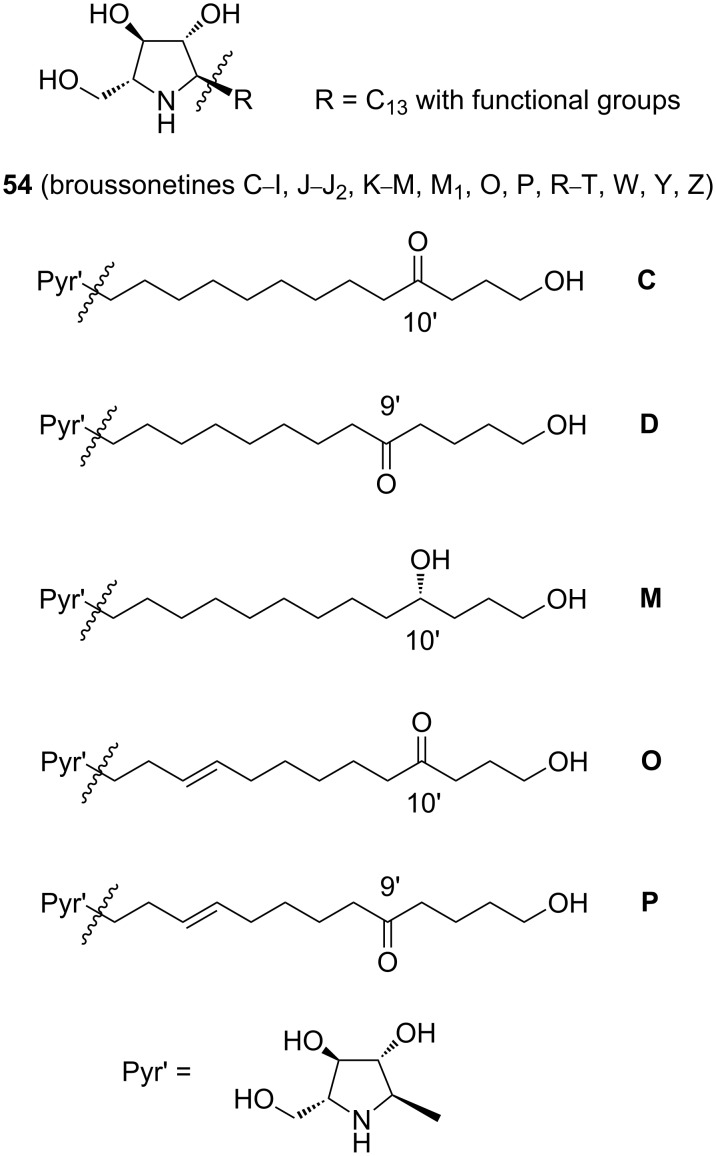
Structural features of broussonetines **54**.

In fact, the case of broussonetines is much more complicated. This subgroup is currently represented by 30 reported examples, all isolated from plant species and used in folk medicine in China and Japan. Most broussonetines display marked inhibitory activities on various glycosidase types, with selectivities differing from that of other standard iminosugars such as DNJ. In the majority of the broussonetines (**54**, Scheme 10), a common polyhydroxylated pyrrolidine building block (possibly prepared via protocols including RCM) is bound to a side chain fragment of 13 C-atoms, diversely functionalized. For the introduction of the appropriate side chain, cross-metathesis appeared to be the most versatile method, permitting access to many members of this family, both naturally occurring and analogues. Two types of metathesis processes, RCM and CM, can be thus advantageously intertwined in the synthesis of broussonetines.

**Scheme 11:**
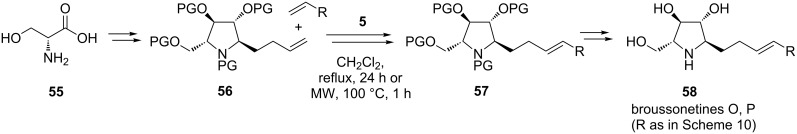
Synthesis of broussonetines by cross-metathesis.

For instance, the syntheses of broussonetines C, D, M, O and P were completed by Falomir, Marco et al. [62–63] in a convergent, stereocontrolled way starting from commercial D-serine (**55**) as the chiral precursor and by applying the critical step of cross-metathesis (the first-ever synthesis of broussonetines O and P) (Scheme 11).

The cross-metathesis reaction was promoted by the 2nd-generation Grubbs catalyst (**5**, in CH_2_Cl_2_, by heating under reflux in a N_2_ atmosphere for 24 h or by heating for 1 h at 100 °C under microwave irradiation). As expected in a cross-metathesis process, a mixture of three products (CM product plus the two homo-metathesis products, all in both stereoisomeric forms) was obtained. Homo-metathesis products from either **56** or the alkene were recycled in the cross-metathesis stage to provide an additional amount of the useful product **57**, thus enhancing the overall yield.

### Piperidine-based iminocyclitols

**Scheme 12:**
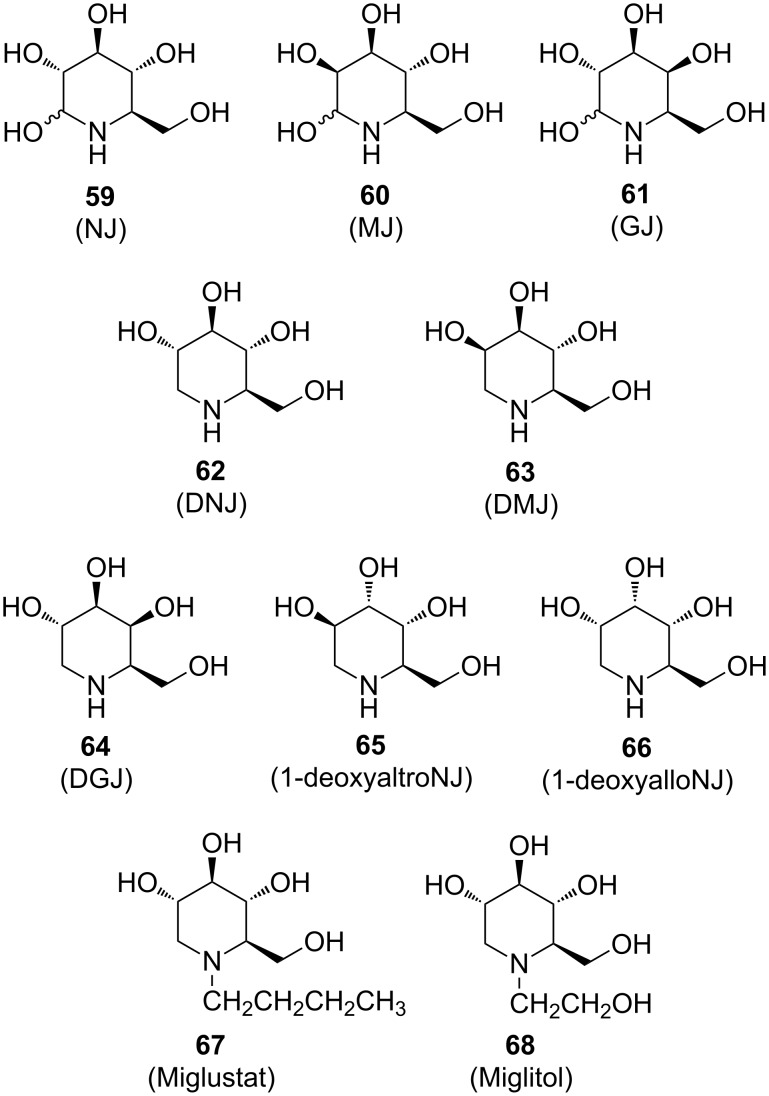
Representative piperidine-based iminocyclitols.

During the last decade, polyhydroxylated piperidines have been the target of much cutting-edge synthesis work [8]. Such compounds are of special interest as therapeutic agents and as tools for the study of cellular mechanisms and metabolic diseases. From this class, nojirimycin (NJ, trivial name for 5-amino-5-deoxy-D-glucopyranose) (**59**), the first alkaloid discovered that mimicks a sugar (originally isolated from *Streptomyces* filtrate but also found in other bacterial cultures and plant sources), is a potent glycosidase inhibitor. In aqueous solution nojirimycin exists in both the α- and β-forms, each of which is responsible for inhibition of α- or β-glucosidase, respectively. Similar to its other congeners, mannonojirimycin (**60**; MJ or nojirimycin B) and galactonojirimycin (**61**; GJ or galactostatin), nojirimycin is unstable because hemiacetal structures can be adopted [8]. 1-Deoxynojirimycin (**62**; DNJ), the more stable 1-deoxy analogue of nojirimycin, represents the main motif of a large family of iminocyclitols (e.g., **63**–**66**). Although numerous other piperidine iminocyclitols have shown encouraging results against HIV and in cancer therapy, the deoxynojirimycin family is certainly the most interesting. Two deoxynojirimycin derivatives have already found clinical applications: *N*-butyl-1-deoxynojirimycin (Miglustat, **67),** in the treatment of type-II diabetes, and *N*-hydroxyethyl-1-deoxynojirimycin (Miglitol, **68**; FDA approved**)** in the treatment of Gaucher’s disease (Scheme 12) [8].

**Scheme 13:**
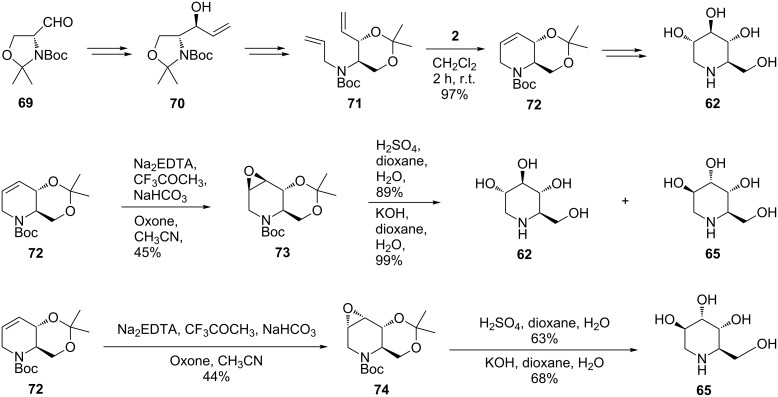
Total synthesis of 1-deoxynojirimycin (**62**) and 1-deoxyaltronojirimycin (**65**).

Takahata et al. [64] exploited RCM for the contruction of the piperidine ring of 1-deoxynojirimycin (**62**) and its congeners (1-deoxymannonojirimycin (**63**), 1-deoxyaltronojirimycin (**65**), and 1-deoxyallonojirimycin (**66**), Scheme 13). In their methodology, the D-serine-derived Garner aldehyde **69** provides an attractive starting point since it reacts with organometallic reagents with a high degree of diastereoselectivity and minimal racemization. N-allylation (allyl iodide/NaH; 95% yield) of an intermediate derived from **70** gave the diolefin product **71,** which was then subjected to RCM (dichloromethane, 20 °C) in the presence of the 1st-generation Grubbs catalyst [(Cl_2_(Cy_3_P)_2_Ru=CHPh)] (**2**) to form the chiral tetrahydropyridine building block **72** in 97% yield. The stereochemistry of **72** was unambiguously confirmed by transformation into the known *trans*-3-hydroxy-2-hydroxymethylpiperidine. The tetrahydropyridine scaffold **72** allowed an efficient synthesis of 1-deoxynojirimycin **62**, and its stereoisomers **65** and **66**. Thus, acid hydrolysis of the epoxy ring in the *anti* isomer **73** (H_2_SO_4_/dioxane/H_2_O, 0.2:3:2) gave 1-deoxynojirimycin (**62**) and 1-deoxyaltronojirimycin (**65**) in a 1:1 ratio and in 89% yield. Conversely, basic cleavage of the epoxide **73** (KOH/dioxane/H_2_O) led preferentially to **65** (1:1.5 ratio **62**/**65**; 99% yield). It should be noted that in the case of the *syn* epoxide **74** both acidic and basic hydrolysis afforded only 1-deoxyaltronojirimycin (**65**), in 63 and 68% yields, respectively.

**Scheme 14:**
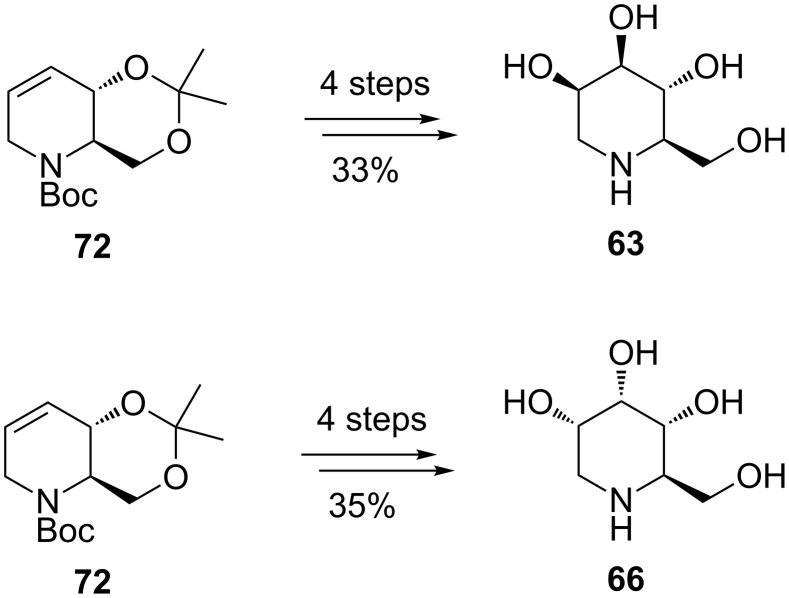
Synthesis by RCM of 1-deoxymannonojirimycin (**63**) and 1-deoxyallonojirimycin (**66**).

Further manipulations based mainly on stereoselective dihydroxylation (K_2_OsO_4_·2H_2_O; NMO as co-oxidant) of the useful building block **72** are at the core of the synthesis of 1-deoxymannonojirimycin (**63**) and 1-deoxyallonojirimycin (**66**) (Scheme 14). Although 1-deoxynojirimycin (**62**) and 1-deoxyaltronojirimycin (**65**) were obtained in a rather selective manner, a similar route to deoxymannojirimycin **(63**) and 1-deoxyallonojirimycin (**66**) did not achieve the same degree of selectivity, presumably due to difficulties in transforming the endocyclic double bond of the RCM product **72** into the targeted *trans* diols. Clean epoxide opening is frequently troublesome, being governed by a number of factors.

**Scheme 15:**
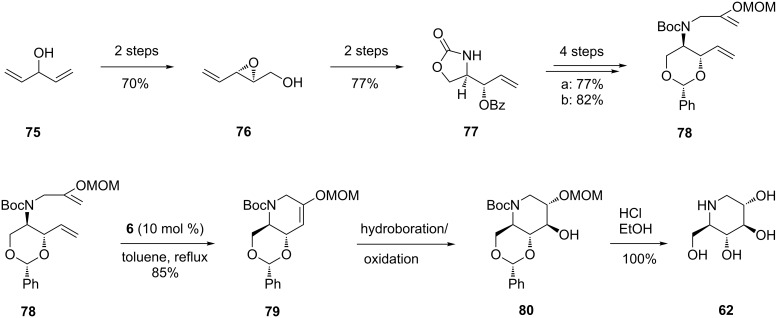
Total synthesis of (+)-1-deoxynojirimycin (**62**).

An improvement in the selectivity and efficiency of the total synthesis of (+)-1-deoxynojirimycin (**62**) (24% overall yield) was made by Poisson et al. [65], who developed a one-pot tandem protocol involving enol ether RCM/hydroboration/oxidation, which gave the best results when the Hoveyda–Grubbs catalyst **6** was used in the RCM (Scheme 15).

Interestingly, in this case the asymmetric synthesis of the protected RCM precursor **78** started from a non-chiral source, the alcohol **75**, and proceeded with complete stereocontrol over the 11 steps involved. All attempts to achieve metathesis on another diene precursor having an endocyclic N-atom (the result of N-alkylation of **77** with 3-iodo-2-(methoxymethyloxy)prop-1-ene) led to either recovery of the starting material or olefin isomerization, even in the presence of a number of ruthenium hydride traps. Satisfactory results in RCM were, however, obtained from **78**: in the presence of the 2nd-generation Grubbs catalyst **5** and benzoquinone in refluxing toluene, **78** was converted into the cyclized enol ether **79** in 70% yield, while with the Hoveyda–Grubbs catalyst (**6**, 10 mol %; benzoquinone 10 mol %; in refluxing toluene) **79** was obtained in 85% yield. The three reaction steps leading from **78** to **80**, i.e., RCM/hydroboration/oxidation, could be accomplished in one-pot to afford the product as a single isomer (all-*trans* triol). The prepared (+)-1-deoxynojirimycin (**62**) displayed spectroscopic data which perfectly matched those of the natural product.

**Scheme 16:**
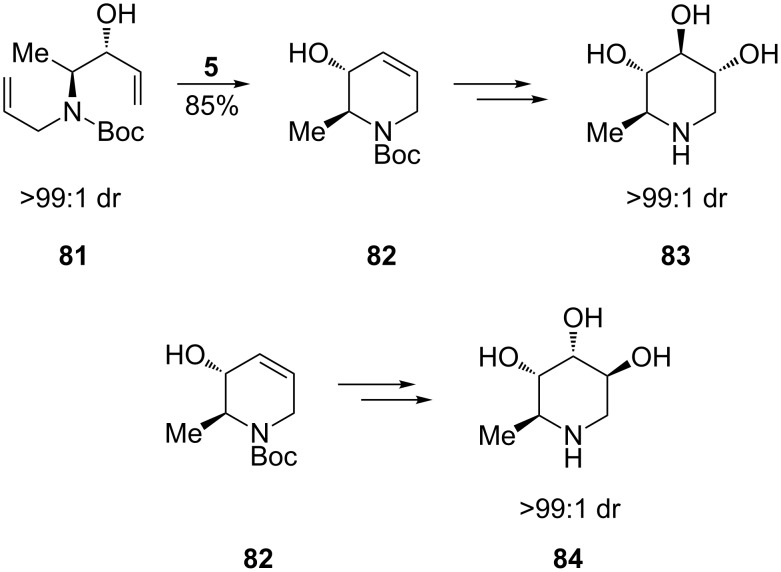
Synthesis of *ent*-1,6-dideoxynojirimycin (**83**) and 5-amino-1,5,6-trideoxyaltrose (**84**).

An important precursor for the synthesis of polyhydroxylated piperidines, (3*R*,4*S*)-3-hydroxy-4-*N*-allyl-*N*-Boc-amino-1-pentene (**81**), could be obtained as a single diastereomer via the addition of vinyl Grignard to the *N*-Boc-*N*-allyl aminoaldehyde, which was derived from the methyl ester of natural, enantiopure L-alanine. Having built the tetrahydropyridine scaffold **82** by RCM of **81** using the 2nd-generation Grubbs catalyst (**5**; 85% yield), Park et al. [66] were able to effect its stereodivergent dihydroxylation, via a common epoxide intermediate, to yield a range of interesting hydroxylated piperidines. This included *ent*-1,6-dideoxynojirimycin (*ent*-1,6-dDNJ, **83**) (28% overall yield from *N*-Boc-L-alanine methyl ester) and 5-amino-1,5,6-trideoxyaltrose (**84**) (29% overall yield from *N*-Boc-L-alanine methyl ester), which were produced with excellent diastereoselectivity (>99:1 dr, Scheme 16). It appears that this total synthesis of *ent*-1,6-dDNJ (**83**) is the most expeditious to date.

**Scheme 17:**
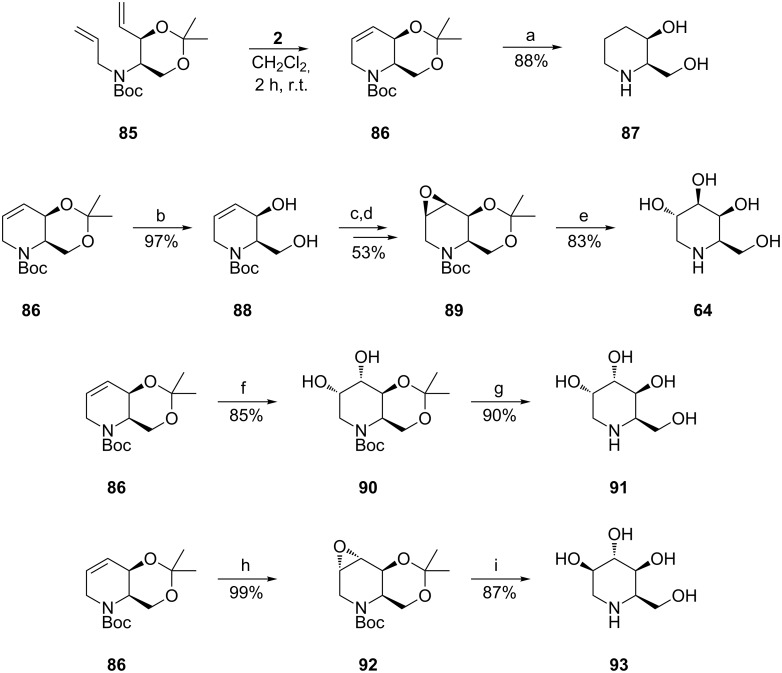
Synthesis of 1-deoxygalactonojirimycin (**64**), 1-deoxygulonojirimycin (**91**) and 1-deoxyidonojirimycin (**93**) [Step c: *m*-CPBA, NaH_2_PO_4_, CH_2_Cl_2_, 0 °C to r.t. Step d: 2,2-dimethoxypropane, PPTS, acetone, r.t. Step e: H_2_SO_4_, 1,4-dioxane, H_2_O, reflux. Step f: K_2_OsO_4_·2H_2_O, NMO, acetone, H_2_O, r.t. Step g: HCl, MeOH. Step h: Oxone, CF_3_COCH_3_, NaHCO_3_, aqueous Na_2_·EDTA, CH_3_CN, 0 °C. Step i: 0.3 M KOH, 1,4-dioxane, H_2_O, reflux].

It was again Takahata et al. [67] who successfully tackled the synthesis of 1-deoxygalactonojirimycin (**64**, DGJ) and its congeners, 1-deoxygulonojirimycin (**91**) and 1-deoxyidonojirimycin (**93**) (Scheme 17), relying in the first step on the diastereoselective addition of a vinyl organometallic reagent to D-Garner aldehyde. Once more, for construction of the piperidine ring in **86**, RCM (1st-generation Grubbs catalyst, **2**) was applied to the prerequisite diene **85** bearing a cyclic conformation constraint. The stereochemistry of the chiral building block **86** was confirmed by conversion into the known compound, *cis*-2-hydroxymethyl-3-hydroxypiperidine (**87**), (step a). For 1-deoxygulonojirimycin (**91**) a highly selective dihydroxylation (step f) was performed on **86**, under Upjohn conditions. For 1-deoxygalactonojirimycin (**64**) and 1-deoxyidonojirimycin (**93**), transformation of **86** proceeded via *syn* (step c) and *anti* (step h) epoxidation of the internal double bond in **86**, respectively, and subsequent hydrolysis.

**Scheme 18:**
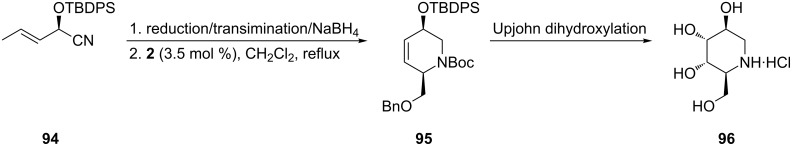
Synthesis of L-1-deoxyaltronojirimycin (**96**).

Quite recently, an interesting synthesis of three 1-deoxynojirimycin-related iminosugars, L-1-deoxyaltronojirimycin (**96**), D-1-deoxyallonojirimycin (**66**), and D-1-deoxygalactonojirimycin (**64**), was reported by Overkleeft et al. to occur from a single chiral cyanohydrin **94**, made available via a chemoenzymatic approach with almond hydroxynitrile lyase (*pa*HNL) [68]. The key steps in the synthetic scheme comprise the cascade Dibal reduction/transimination/NaBH_4_ reduction of the enantiomerically pure **94**, followed by the RCM step (CH_2_Cl_2_, 3.5 mol % 1st-generation Grubbs catalyst, reflux under Ar for 48 h) and Upjohn dihydroxylation to afford the target compounds (Scheme 18 for **96**).

**Scheme 19:**
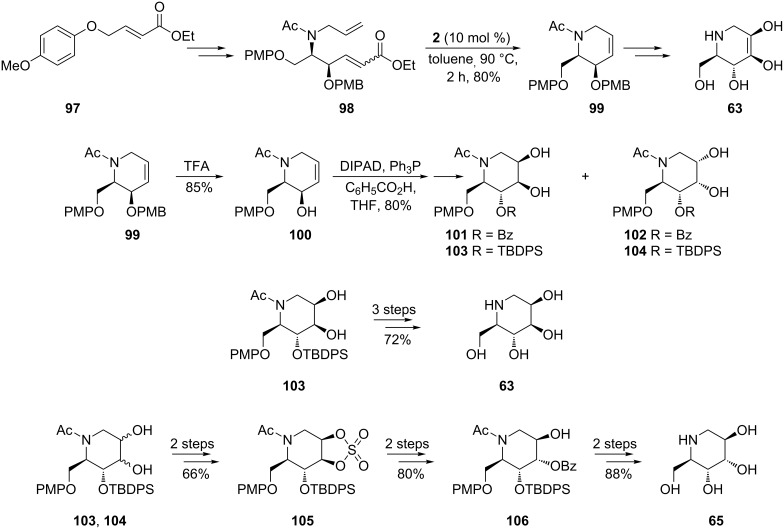
Synthesis of 1-deoxymannonojirimycin (**63**) and 1-deoxyaltronojirimycin (**65**).

RCM promoted by the 1st-generation Grubbs catalyst **2** is starring again in the divergent, flexible methodology disclosed by Singh and Han [69] for the asymmetric synthesis of several deoxyiminocyclitols (1-deoxymannonojirimycin (**63**), 1-deoxyaltronojirimycin (**65**), 1-deoxygulonojirimycin (**91**), 1-deoxyidonojirimycin (**93**), Scheme 19). Ingeniously selecting as starting material the achiral olefin **97**, suitable for electronic and aryl–aryl stacking interactions with the regioselective osmium catalyst, they conducted asymmetric aminohydroxylation (regioselectivity >20:1; enantioselectivity >99% ee) in good yield (70%) to get the RCM precursor diene **98**, appropriately protected. Under common RCM conditions (10 mol % 1st-generation Grubbs catalyst **2**, toluene, 90 °C, 2 h) **98** was then converted to the key cyclo-olefin intermediate **99** (80% yield). From the latter, the targeted iminocyclitols **63** and **91** have been obtained after artful manipulation of routine protocols (diastereoselective dihydroxylation and protection/deprotection). To access 1-deoxyaltronojirimycin (**65**) and 1-deoxyidonojirimycin (**93**), introduction of an additional step involving a cyclic sulfate was necessary.

**Scheme 20:**
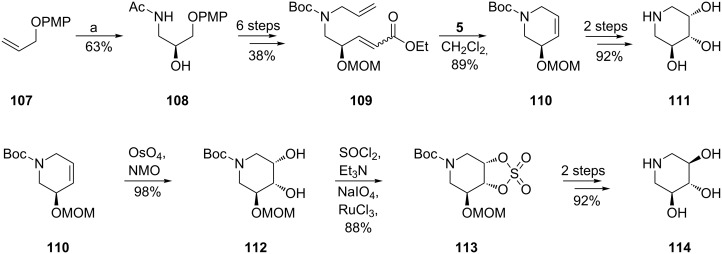
Synthesis of 5-des(hydroxymethyl)-1-deoxymannonojirimycin (**111**) and 5-des(hydroxymethyl)-1-deoxynojirimycin (**114**).

A similar methodology was used by Han [70] to prepare 5-des(hydroxymethyl)-1-deoxynojirimycin (**114**) and its mannose analogue **111** (as HCl salts) in a highly stereoselective mode starting from a different common olefin, **107** (Scheme 20). In this case, RCM was promoted by the 2nd-generation Grubbs catalyst **5** which ensured a high yield of the ring closure (89%) under milder conditions (CH_2_Cl_2_): all attempts to employ the 1st-generation Grubbs catalyst **2** in RCM failed, supposedly because of an unfavourable steric environment during generation of the Ru–carbene species from **109,** as compared to **98** (distinct N-protective groups). Cyclic sulfate chemistry was again invoked for effectively performing the synthesis of **114**.

**Scheme 21:**
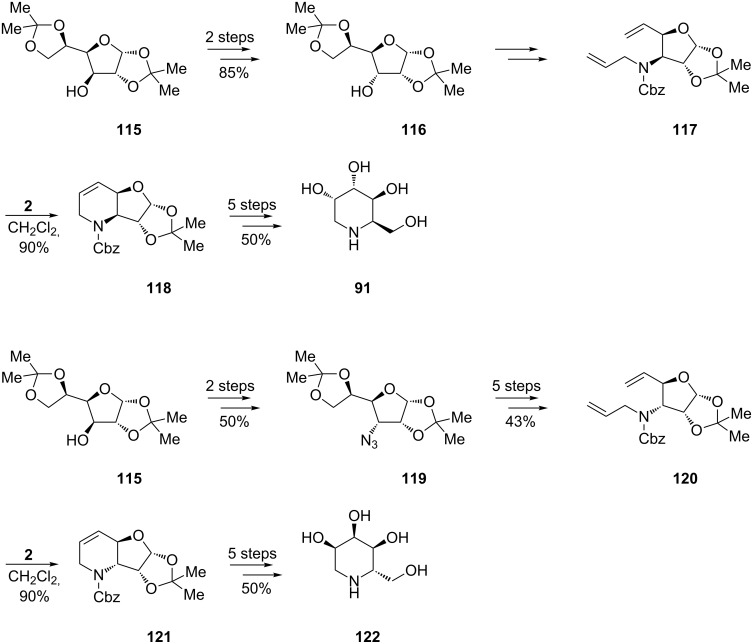
Synthesis of D-1-deoxygulonojirimycin (**91**) and L-1-deoxyallonojirimycin (**122**).

Introducing a genereal strategy for synthesis of deoxyazasugars based on cheap D-glucose, Ghosh et al. also laid groundwork for the preparation of D-1-deoxygulonojirimycin (**91**) (previously communicated by Takahata [67]; Scheme 17) and L-1-deoxyallonojirimycin (**122**) (Scheme 21) starting from protected diacetone glucose **115** [71]. Different pathways were devised for **91** and **122** via the epimeric RCM precursors **117** and **120**, respectively. High yielding cyclization of these dienes, in the presence of the 1st-generation Grubbs catalyst **2** (10 mol %, in CH_2_Cl_2_, under argon, 24 h at 50 °C), led to **118** and **121** with preserved configurations at the stereogenic centre, which therefore allowed the desired stereochemistry in the isomeric final products **91** and **122**.

D-Fagomine (1,2,5-trideoxy-1,5-imino-D-arabinitol or 1,2-dideoxynojirimycin) (**129**) a natural iminosugar present in buckwheat (widely used in traditional recipes) is an efficient agent for preventing sharp blood glucose peaks after the intake of refined carbohydrates and for positively influencing intestinal microbiota by favouring adhesion of probiotics. It is supposed that fagomine enhances glucose-induced insulin secretion by accelerating the processes which follow glyceraldehyde 3-phosphate formation in the glycolytic pathway.

**Scheme 22:**
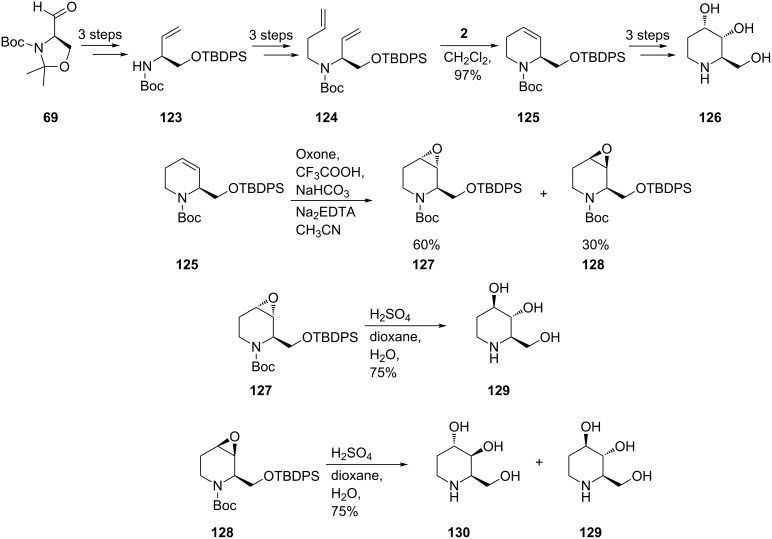
Total synthesis of fagomine (**129**), 3-*epi*-fagomine (**126**) and 3,4-di-*epi*-fagomine (**130**).

The total synthesis, involving RCM, of fagomine (**129**) and its congeners 3-*epi*-fagomine (**126**) and 3,4-di-*epi*-fagomine (**130**) was achieved by Takahata et al. [72] based again on the Garner aldehyde **69** derived from D-serine. To construct the chiral 1,2,5,6-tetrahydropyridine core **125**, the authors resorted to catalytic ring-closing metathesis induced by the 1st-generation Grubbs catalyst **2**, with subsequent stereoselective dihydroxylation (under modified Upjohn conditions, Scheme 22). For iminocyclitols containing *trans* diols at the 3- and 4-positions an epoxy functionality at the double bond in **125** was introduced. While the *syn* epoxide **128** led to a mixture of fagomine (**129)** and 3,4-di-*epi*-fagomine (**130**), the *anti* epoxide **127** gave **129** selectively. The 3-*epi*-fagomine (**126**) could also be obtained from the RCM product **125** (by conventional dihydroxylation/deprotection; 10 steps from Garner’s aldehyde **69**).

**Scheme 23:**
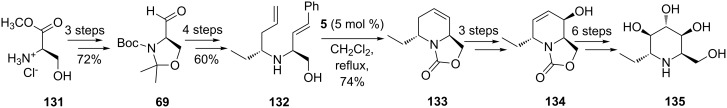
Total synthesis of (+)-adenophorine (**135**).

Adenophorine (α-1-deoxy-1-*C*-methylhomonojirimycin) is a further important iminocyclitol in whose synthesis RCM proved helpful. (+)-Adenophorine (**135)**, a naturally occurring iminocyclitol with a lipophilic substituent at the anomeric position, is active on α-glucosidase which is a valid proof that α-alkylation at C1 does not supress the glycosidase inhibitory effect. Its lack of activity on β-galactosidase once again indicates that the relative position of hydroxy substituents is critical for selectivity. In the seminal work by Lebreton and coworkers [73], the first asymmetric total synthesis of (+)-adenophorine was achieved in 14 steps (3.5% overall yield, Scheme 23), starting from the Garner’s aldehyde **69**. RCM is essential for construction of the 6-membered N-heterocycle in **133.** Protection of the amino alcohols *trans*-**132** and *cis*-**132**, as the corresponding *trans* and *cis* oxazolidinones, afforded a mixture of diastereomers that were not separable on silica gel. After effecting RCM (2nd-generation Grubbs catalyst **5,** 5 mol %) on this mixture, separation of the diastereomers by flash chromatography was possible, affording the pure tetrahydropyridine derivative *trans*-**133** in 74% yield. Successive epoxidations on enantiopure *trans-***133** and then **134**, followed each time by regioselective epoxide opening (with a selenium–boron complex and water, respectively), gave finally **135** with good stereoselectivity. This overall synthesis demonstrates rigorous control at every stage of both the steric configuration of the starting materials and the steric effects induced by substituents attached to the piperidine moiety.

**Scheme 24:**
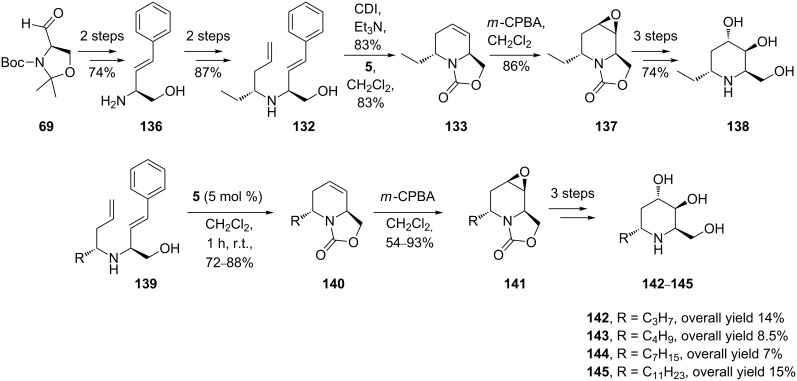
Total synthesis of (+)-5-deoxyadenophorine (**138**) and analogues **142**–**145**.

Related studies by Lebreton et al. [74–76] explored the synthesis of a panel of 6-alkyl substituted piperidine iminocyclitols that had been previously isolated by Asano and coworkers [77] from *Adenophora* spp. These natural products display an unusual structure in that they possess a hydrophobic substituent such as a undecyl, heptyl, butyl or ethyl group at the α position of 1-C. The strategy for (+)-5-deoxyadenophorine (**138**) and analogues **142**–**145** began again from D-Garner aldehyde **69** and also used the powerful RCM as the key step (catalyst **5**, 5 mol %; CH_2_Cl_2_, reflux 1 h) for building the chiral *trans*-2,6-disubstituted-1,2,5,6-tetrahydropyridine scaffold (72–88% yield, Scheme 24).

### Azepane-based iminocyclitols

Iminocyclitols incorporating the azepane ring system are more flexible than the parent pyrrolidine and piperidine iminosugars, and they adopt quasi-flattened, low-energy conformations which can potentially lead to a more favourable binding with the active site of enzymes. The unusual spatial distribution of the hydroxy groups in these compounds should generate new inhibitory profiles. According to in vitro assays, seven-membered ring iminocyclitols are noted inhibitors of α-mannosidase, an enzyme that plays important roles in glycoprotein biosynthesis. Derivatives of this class bearing hydroxymethyl groups at C-6 have been shown to inhibit powerfully lysosomal α-mannosidase while displaying varying potencies toward α-1,6-mannosidase. On the other hand, N-alkylated polyhydroxylated azepanes with the D-glucose or L-idose configuration proved to be potent β-glucosidase inhibitors that showed only weak activity towards α-glucosidase and α-mannosidase [78–80]. Malto-oligosaccharides and analogues of di- and trisaccharides containing polyhydroxylated azepane moieties are glucosidase or HIV/FIV-protease blockers, or both.

**Scheme 25:**
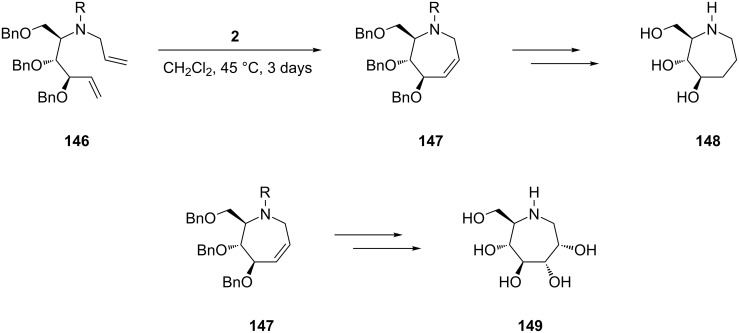
Synthesis by RCM of 1,6-dideoxy-1,6-iminoheptitols **148** and **149**.

As for the previous classes, in the synthesis of seven-membered iminocyclitols RCM provides a focal point in ring closure being responsible for constructing the azepane framework. For example, 1,6-dideoxy-1,6-iminoheptitols **148** and **149**, that can be viewed as higher homologues of fagomine and nojirimycin, respectively, are easily accessed from the protected diene **146**. RCM of this diene with 1st-generation Grubbs catalyst (**2**, CH_2_Cl_2_, 45 °C) gives the common N-heterocyclic intermediate **147** (91% yield, Scheme 25). Hydrogenation of the latter gives the iminocyclitol **148** whereas its *cis*-selective dihydroxylation affords the pentahydroxy derivative **149**.

**Scheme 26:**
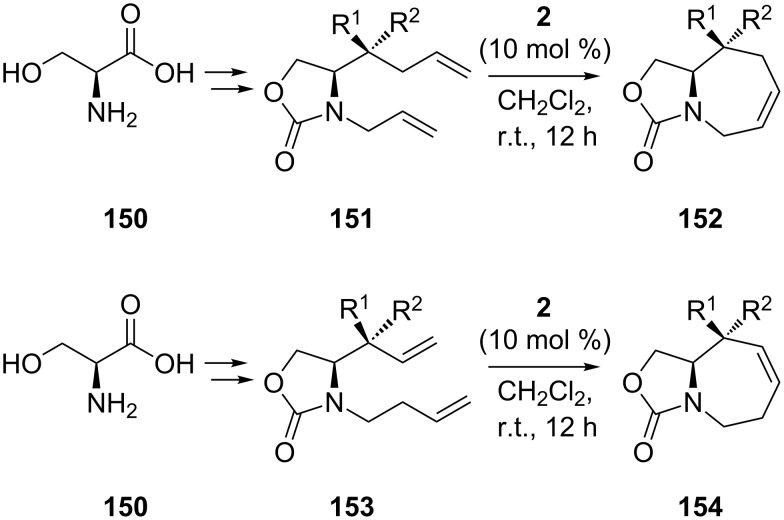
Synthesis by RCM of oxazolidinyl azacycles **152** and **154**.

Starting from L-serine **150**, Lin et al. [81] devised a refined method for the synthesis of structurally diverse stereoisomers of polyhydroxyazepanes. In their complex strategy, RCM (1st-generation Grubbs catalyst, 10 mol %, CH_2_Cl_2_, reflux, 12 h) plays a significant role by leading to a panel of oxazolidinyl azacyclic products (e.g., **152** and **154**). Remarkably, the authors expertly arranged the positions of the double bonds involved in RCM on the one hand by addition of alkenyl nucleophiles (with different lengths) on aldehyde intermediates, and on the other hand by placing the second double bond at a different distance relative to the nitrogen atom (Scheme 26).

**Scheme 27:**
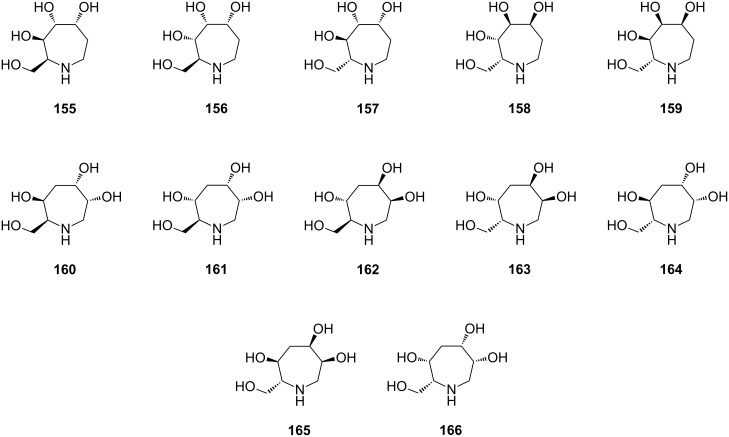
Representative azepane-based iminocyclitols.

There are two advantageous follow-ups: (i) a desired location of the double bond in the azacyclic RCM product, and therefore of the hydroxyls in the final iminocyclitol products, and (ii) possible extension of the methodology to the construction of other ring sizes (5- to 8-membered). This versatile approach, featuring the basic sequence metathesis/dihydroxylation, led in good yields to a number of stereoisomers of seven-membered iminocyclitols exhibiting glycosidase inhibitory properties (Scheme 27). Of the compounds shown in Scheme 27, compound **161** with L-configuration at C-6 exhibited the highest inhibition.

**Scheme 28:**
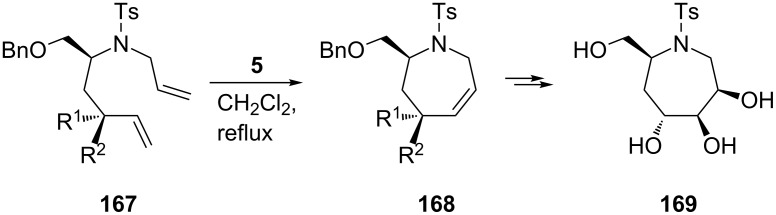
Synthesis of hydroxymethyl-1-(4-methylphenylsulfonyl)azepane 3,4,5-triol (**169**).

As illustrated in Scheme 28, the 2nd-generation Grubbs catalyst **5** found further application in the recent synthesis of seven-membered ring iminocyclitols, e.g., of 7-hydroxymethyl-1-(4-methylphenylsulfonyl)azepane-3,4,5-triol (**169**). This compound shares a common configuration of the hydroxy groups with its lower cyclic homologue, 1-deoxymannojirimycin (DMJ, **63**), a selective inhibitor of α-mannosidase I [82].

**Scheme 29:**
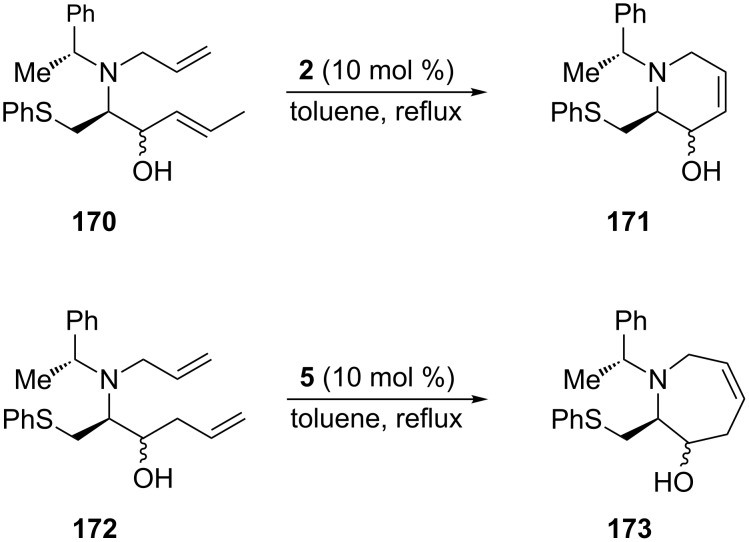
Synthesis by RCM of tetrahydropyridin-3-ol **171** and tetrahydroazepin-3-ol **173**.

Lee et al. [83] also used RCM induced by the 1st-generation Grubbs catalyst **2** or the 2nd-generation Grubbs catalyst **5** (10 mol %, reflux in toluene; 90–91% yield) in an efficient approach to targeted enantiomerically pure, stereochemically defined, six- and seven-membered heterocyclic scaffolds, i.e., the tetrahydropyridin-3-ol **171** and tetrahydroazepin-3-ol **173** (Scheme 29).

These diversely substituted N-heterocyclic compounds, endowed with an internal double bond, are versatile precursors suitable for further functionalization. Asymmetric syntheses employing such intermediates could lead to disclosure of further biologically relevant piperidine/azepane alkaloids and iminosugars.

## Conclusion

The paper introduces the broad scope of olefin metathesis as a key reaction in synthetic strategies for the preparation of monocyclic iminocyclitols. In comparison with earlier well-established protocols, olefin metathesis (RCM, CM) offers shorter, simpler and atom-economical routes, and preserving at the same time the carefully designed and worked for stereochemistry of the precursors. Whereas RCM is the method of choice for constructing the pyrrolidine, piperidine or azepane cores of monocyclic iminocyclitols, CM rewardingly permits access to a collection of new iminocyclitols simply by using one heterocyclic intermediate endowed with an olefinic side-chain and changing only its olefin partner. The reaction conditions applied in these crucial steps are rather conventional for metathesis processes, with the choice of the temperature and solvent (refluxing CH_2_Cl_2_ or toluene) being dictated by steric demands, and hence energetics, for ring-closing or cross-coupling. While the 1st- and 2nd-generation Grubbs catalysts (5–10 mol %) are the catalysts most frequently employed, the 2nd-generation Grubbs and Hoveyda–Grubbs catalysts perform better when harsher conditions are required. Despite the various functionalities existing on the metathesis precursors and products, sensitive metathesis catalysts are quite productive due to inventive protection/deprotection at the O- and N-heteroatoms. Such delicate operations are skillfully conceived so as to either maintain or reverse the geometry at stereogenic centres, as required. In the ensemble of stereocontrolled reactions concentrating on the economical achievement of the targeted number and relative positions of hydroxy, hydroxyalkyl or other substituents, i.e., the overall structure that hinges on the biological activity, metathesis is surely a fine addition which is bound to succeed in creating novel azasugars with a larger therapeutic window. The metathesis approach may ultimately yield benefits for patients suffering from metabolic disorders, cancer and viral diseases.
